# Rituximab‐induced serum sickness in immunobullous disorders: A case series

**DOI:** 10.1002/ccr3.9152

**Published:** 2024-07-07

**Authors:** Mehdi Gheisari, Toktam Safari Giv, Elnaz Pourgholi, Shirin Zaresharifi

**Affiliations:** ^1^ Skin Research Center Shahid Beheshti University of Medical Sciences Tehran Iran; ^2^ Department of Dermatology, Loghman‐Hakim Hospital, School of Medicine Shahid Beheshti University of Medical Sciences Tehran Iran

**Keywords:** immunobullous disorders, pemphigus vulgaris, rituximab, serum sickness

## Abstract

**Key Clinical Message:**

Rituximab‐induced serum sickness (RISS) is a rare complication of Rituximab (RTX) in immunobullous disorders. Clinicians should be aware of the occurrence of serum sickness symptoms during RTX administration, and prompt initiation of corticosteroid therapy is crucial in these patients. Additionally, RISS may occur with subsequent RTX doses and patients should be counseled accordingly.

**Abstract:**

Rituximab (RTX) is a chimeric monoclonal anti‐CD20 antibody which has gained approval for the treatment of various autoimmune and lymphoproliferative disorders. While RTX‐induced minor reactions, including immediate infusion‐related reactions, are common, serum sickness is rare. Limited data exist regarding rituximab‐induced serum sickness (RISS) in pemphigus vulgaris (PV) and mucous membrane pemphigoid (MMP). We report two cases of RISS following RTX administration in PV and MMP patients. Both patients presented with typical symptoms of serum sickness after RTX infusion, necessitating drug cessation and corticosteroid therapy for resolution. RISS represents a rare complication of RTX therapy. Clinicians should maintain awareness of serum sickness presentations during and post‐RTX administration.

## 1 INTRODUCTION

Rituximab (RTX) is an anti‐CD20 monoclonal antibody that targets mature B cells.^1^ It is an FDA‐approved drug for the management of autoimmune and lymphoproliferative disorders like Rheumatoid Arthritis (2), Pemphigus Vulgaris (PV), CD20 positive B‐cell Non‐Hodgkin's Lymphoma, and Chronic Lymphocytic Leukemia (CLL).^2^, ^3^, ^4^ The off‐label use of this treatment has also demonstrated efficacy in managing moderate to severe cases of mucous membrane pemphigoid (MMP).^5^

Immediate infusion‐related reactions including fever, cough, and dyspnea have been mentioned as a result of immunologic response. Agranulocytosis and severe infection are the most commonly reported late complications.^6^, ^7^ Serum sickness manifests as a Type III hypersensitivity reaction, mediated by immune complexes, whereby these complexes are deposited on endothelial tissue, instigating an inflammatory cascade that induces symptoms including rash, arthritis, arthralgia, and other systemic manifestations. The onset of this reaction necessitates the confluence of the antigen alongside antibodies specific to it, culminating in the formation of antigen–antibody complexes.^8^ By producing human anti‐chimeric antibodies, RTX causes serum sickness.^9^, ^10^ Limited data exist about serum sickness in patients with PV and MMP. We report two cases with PV and MMP who developed RISS.

## 2 CASE HISTORY

### 2.1 Case 1

A 42‐year‐old female presented with vesiculobullous lesions affecting the chest, abdomen, and upper extremities, as well as erosive lesions on lips, oral, and genital mucosa persisting for 8 weeks, culminating in a diagnosis of PV based on histopathology and direct immunofluorescence (DIF). Oral prednisolone 40 mg/day and intravenous RTX infusion were prescribed.

Infusion of 500 mg of RTX in 500 mL of 5% dextrose solution over 4–6 h was done uneventfully after normal results of preliminary investigations. The second infusion was given one week later and no adverse reaction was reported during the infusion.

After 7 days of the second infusion, the patient was referred to the hospital with general malaise, myalgia, and fever along with pain and swelling of small joints of hands as well as both ankles and knees. Physical examination revealed febrile status (T = 38.5 axillary), and tenderness and swelling of the proximal interphalangeal (PIPs), distal interphalangeal (DIPs) joints; and both ankles and knees. No new skin rash or lymphadenopathy was detected.

### 2.2 Case2

A 35‐year‐old woman presented with an 11‐month history of painful gingival erosions. Despite multiple courses of antibiotic treatment, her lesions continued to worsen. She was referred to our clinic, where we performed punched biopsies for histopathologic and direct immunofluorescent evaluation. Light microscopy revealed a subepidermal blister and a dermal infiltrate composed of lymphocytes, histiocytes, and variable numbers of neutrophils, eosinophils, and plasma cells. Direct immunofluorescence microscopy of normal‐appearing perilesional tissue demonstrated linear continuous deposits of IgG, IgA, and C3 at the dermoepidermal junction. Salt‐split skin indirect immunofluorescence revealed IgG, IgA, and C3 binding to both the epidermal and dermal sides of the split. Physical examination did not show involvement of other mucous membranes or the skin. Based on the clinicopathological evaluations the diagnosis of MMP was made and dapsone was initiated at 50 mg daily and titrated up to 100 mg daily. However, after 2 months due to a suboptimal response to Dapsone therapy, RTX was initiated. One week following the first dose of RTX, the patient reported experiencing fever, severe arthralgia, and pruritus morbilliform rashes on her extremities. The patient's symptoms were alleviated after taking analgesics, and she did not report these symptoms during her subsequent visit.

Upon seeking a subsequent dose of RTX without disclosing the prior adverse events, the patient experienced a recurrence of severe arthralgia and myalgia, accompanied by fever and polyarthritis, generalized maculopapular skin rashes, arthritis, and a pulse rate of 110 and respiratory rate of 20.

## 3 DIFFERENTIAL DIAGNOSIS, INVESTIGATIONS, AND TREATMENT

The differential diagnosis of serum sickness includes viral exanthems, urticarial vasculitis, vasculitis, sepsis, and other types of drug reactions.

### 3.1 Case 1

Laboratory examinations and imaging were conducted, revealing soft tissue swelling on x‐rays of the hand joints, elevated C‐reactive protein (CRP) and erythrocyte sedimentation rates (ESR) but normal results on complete blood count (CBC), renal function tests, liver function tests, urine analysis, and rheumatological lab tests.

**FIGURE 1:**
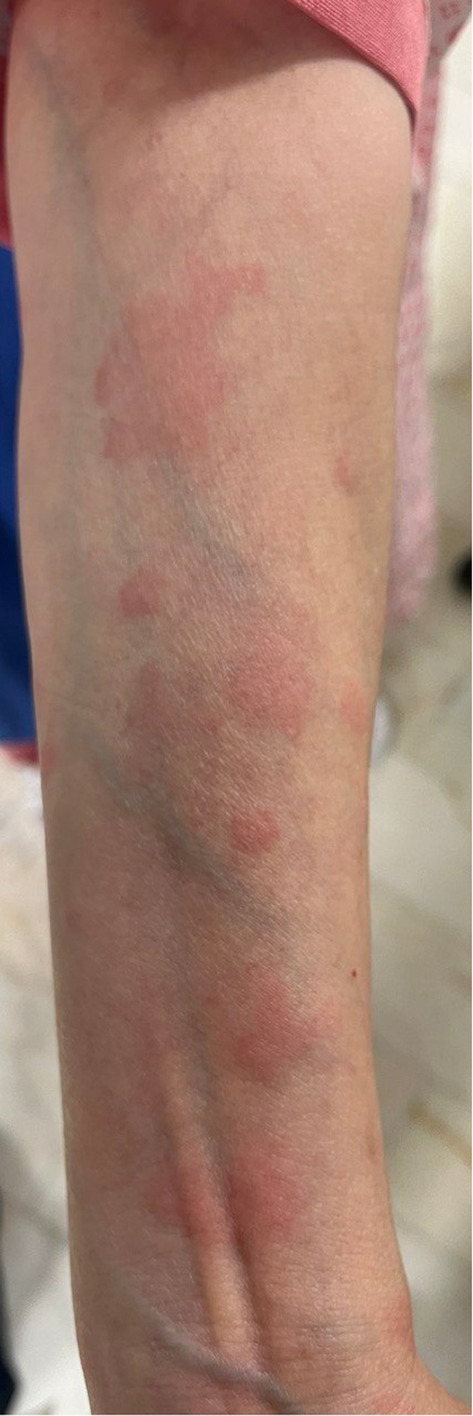
Urticarial lesions due to serum sickness in a pemphigus vulgaris patient receiving rituximab.

Broad‐spectrum antibiotics (intravenous imipenem 500 every 6 h and intravenous vancomycin 1 g twice daily) were initiated due to suspected sepsis. On the sixth day of admission, after stabilizing the patient's condition the third dose of 500 mg RTX was initiated but, after half an hour of starting the infusion, she developed dyspnea, swollen lips, and generalized skin rash. Physical examination revealed tachypnea, tachycardia, and blanchable maculopapular erythematous rash on the trunk, extremities, and face (Figure 1). The infusion was halted, and 100 mg of hydrocortisone was administered intravenously, leading to symptom resolution within approximately 2 h. The patient was diagnosed with a RISS based on the history, physical examination, and excluding other differential diagnoses. She was treated with oral prednisolone 40 mg and analgesic agents for arthritis and myalgia. The platelet count started to decrease from 125,000 to 25,000 platelets per microliter (mcL) (normal range: 150,000–450,000 per mcL) on serial CBCs after the last infusion of RTX. Due to hematologic consultation and normal peripheral blood smear (PBS), the reduction of platelets was attributed to the serum sickness. One week later, she was discharged with advice to avoid RTX in the future.

### 3.2 Case2

The infusion of RTX was halted, and 100 mg of hydrocortisone was administered. Subsequently, the patient was prescribed oral prednisolone 40 mg and analgesics. Following a four‐day hospitalization period, she was discharged in stable condition, with guidance to abstain from further RTX treatment.

## 4 OUTCOME AND FOLLOW‐UP

Following an 18‐month follow‐up period, in Case 1, the patient remained asymptomatic without requiring corticosteroids or corticosteroid‐sparing agents.

In Case 2, 40 mg of prednisolone was gradually tapered over a span of 3 months. However, after 1 year, due to a recurrence of oral lesions, prednisolone 30 mg dosage was prescribed again and mycophenolate mofetil was initiated at a dosage of 1 gram twice daily. The prednisolone dosage was further tapered over 8 weeks. The patient is currently on mycophenolate mofetil at a dosage of 1 gram twice daily and prednisolone at 5 mg daily, and her MMP remains controlled.

## 5 DISCUSSION

In this study, we present two cases of RISS in patients with PV and MMP.RTX is an anti‐CD‐20 antibody that induces cell death through various mechanisms, including complement‐mediated cytotoxicity, antibody‐dependent cell‐mediated cytotoxicity, and antibody‐dependent phagocytosis in CD‐20 positive B‐cells.^11^, ^12^ Despite its efficacy, RTX therapy is associated with a spectrum of adverse effects, with serum sickness being a rare but notable complication. Our cases add to the limited data on RISS in PV and MMP patients.

MMP is a heterogeneous type of chronic and autoimmune disorders that are presented as subepithelial blistering involvement of mucous membranes and the skin.^5^ This treatment, although used off‐label, has proven effective in managing moderate to severe cases of MMP.^13^ Lamberts et al., evaluated the safety and effectiveness of RTX in patients with recalcitrant pemphigoid. Twenty‐eight patients with pemphigoid disorders were evaluated. Only one case (the exact subtype of pemphigoid disease was not determined) demonstrated possible symptoms of RISS.^14^ Based on our knowledge, this is the only reported case of RISS in an MMP patient.

While precise incidence data for RISS are lacking, Karmacharya et al. conducted a systematic review and identified only 33 reported cases, with the majority occurring in patients with rheumatologic diseases, notably Sjögren's syndrome, but none in PV patients. About 50% of cases had a classic triad of serum sickness including arthralgia, rash, and fever.^15^ Both of our cases had a typical triad of serum sickness. One case developed the symptoms after the first dose and one after the second dose, aligning with the typical onset timeframe reported in the literature. In most cases, including ours (4 out of 5 patients), corticosteroids were the mainstay of treatment for RISS.^15^

Tavakolpour et al. systematically reviewed 1085 PV cases treated with RTX over 16 years, finding no instances of RISS, further underscoring its rarity in PV.^6^

The majority of RISS occurred in the context of autoimmune diseases rather than malignancies.^16^ Furthermore, the results of the systematic review and the French National study show that RISS occurs more frequently in people aged 38–37.^15^, ^16^

Table 1 presents the details and comparison of RISS in PV that were reported previously, in addition to the current study. There have been 4 other case reports representing RISS sickness in PV patients.^17^, ^18^, ^21^, ^22^ The mean age of RISS occurrence in PV cases was 37 ± 12.21 years. All described cases had similar presentations. All reactions happened during the first or second dose of RTX administration, and the dosage of RTX administered was nearly identical. There were no immediate reactions, and all reactions were delayed.

**TABLE 1:** Details and comparison of RISS among PV cases.

Study	Age/sex	History of treatment	Dosing protocol (amount, dose)	RTX dose number (Cycle number, dosing number in the cycle)	RISS onset after last RTX dose (days)	Immediate transfusion reaction	Lab data	Treatment
Awal 2018^17^	55/F	Dexamethasone‐cyclophosphamide + oral corticosteroids	1000 mg, 2	1, 1	11	No	↑ ESR ↑ CRP	Systemic Corticosteroids Anti‐Inflammatory Drugs
Deshpande 2019^18^	17/M	Prednisolone 40 mg/day	500 mg, 6	1, 6	10	No	↑ ESR ↑ CRP	Prednisolone 30 mg
Krooks 2019^19^	38/F	Fluocinonide (0.05%) topical	1000 mg, 2	1, 1	12	No	↑ CRP	Diphenhydramine Cetirizine
Khatib 2020^20^	33/M	IVIG azathioprine prednisolone	1000 mg, 2	1, 2	11	No	↑ CRP Leukocytosis	Methylprednisolone (1 mg/Kg/Day) Analgesia Paracetamol, Morphine, Fentanyl Patches
Current study	42/F	Prednisolone 40 mg/day	500 mg, 4	1, 2	7	No	↑ ESR ↑ CRP	Prednisolone 40 mg

Abbreviations: CRP, C‐reactive protein; ESR, erythrocyte sedimentation rates; F, female; IVIG, intravenous Immunoglobulin; M, male; PV, pemphigus vulgaris; RISS, rituximab induced serum sickness; RTX, rituximab.

Table 2 shows the clinical symptoms of the RISS patients.^17^, ^18^, ^21^, ^22^ Polyarthralgia, fever, and skin involvement are the most common symptoms. Less frequent symptoms included lethargy, conjunctivitis, and digestive disorders. RISS symptoms are usually modest and self‐limited and the treatment is primarily symptomatic. Serious adverse reactions including glomerulonephritis or neurological sequelae, are rare.^15^

**TABLE 2:** Comparison of the clinical characteristics of serum sickness.

Study	Fever	Malaise	Polyarthralgia or arthritis	Cutaneous manifestations	Conjunctivitis	Digestive disorders
Awal et al. 2018^17^	+	−	+	+	−	−
Deshpande et al. 2019^18^	+	−	+	‐	+	Epigastric pain and vomiting
Krooks et al. 2019^19^	−	+	+	+	−	Abdominal pain
Khatib et al. 2020^20^	+	−	+	+	−	−
Current study	+	+	+	+	−	−

In the mentioned PV cases, RISS did not result in mortality; however, our patient experienced a hemodynamic collapse episode during the third RTX infusion. In patients with severe presentations, some researchers have recommended using plasmapheresis. However, it is challenging to evaluate the benefits of any treatment without a standard placebo group, especially given that serum sickness typically resolves on its own.^20^ Due to the reaction that happened in the first series of RTX infusions in the mentioned PV and RISS cases, the history of RISS is not a useful factor for predicting this reaction. RISS may be presented even in patients who received alternative monoclonal Anti‐CD20.^19^, ^23^ In RISS patients, it is so important to advise against re‐injection of RTX and educate the patient about this complication.

While rheumatological tests often remain unremarkable in RISS cases, contradicting the autoimmune hypothesis, further research into RISS pathophysiology is warranted.^15^

While comprehensive data regarding the relative severity of RISS cases is currently lacking, it appears that akin to other types of serum sickness, the intensity of symptoms is contingent upon dosage. Typically, antigen–antibody complexes are cleared by the mononuclear phagocyte system. However, as the functionality of this system diminishes or becomes inundated by a load of immune complexes, there is a heightened propensity for excessive accumulation of these complexes within the circulation. This culminates in their deposition in tissues or direct formation within affected tissues, consequently precipitating a more pronounced inflammatory reaction.^8^

Owing to the nonspecific symptoms and signs of serum sickness, it can be readily misdiagnosed as other conditions. This was exemplified in our two cases, where the nonspecific nature of the serum sickness symptoms initially led to misattribution to alternative causes. Furthermore, the patient's failure to report these symptoms in subsequent visits resulted in repeated exposure to rituximab infusion. It appears that serum sickness is often underreported due to its indistinct presentation.

## AUTHOR CONTRIBUTIONS

**Mehdi Gheisari:** Supervision; writing – review and editing. **Toktam Safari Giv:** Conceptualization; data curation; writing – original draft; writing – review and editing. **Elnaz Pourgholi:** Data curation. **Shirin Zaresharifi:** Conceptualization; data curation; project administration; writing – review and editing.

## FUNDING INFORMATION

No funding was received for this study.

## CONFLICT OF INTEREST STATEMENT

The authors declare that they have no conflicts of interest.

## CONSENT

Written informed consent has been taken from both patients separately regarding publishing the manuscript.

## Data Availability

The data that support the findings of this study are available from the corresponding author upon reasonable request.

## References

[1] Martin LK , Werth VP , Villaneuva EV , Murrell DF . A systematic review of randomized controlled trials for pemphigus vulgaris and pemphigus foliaceus. J Am Acad Dermatol. 2011;64(5):903‐908. 21353333 10.1016/j.jaad.2010.04.039 PMC7382895

[2] Delate T , Hansen ML , Gutierrez AC , Le KN . Indications for rituximab use in an integrated health care delivery system. J Manag Care Spec Pharm. 2020;26(7):832‐838. 32584674 10.18553/jmcp.2020.26.7.832 PMC10391100

[3] Hanif N , Anwer F . Rituximab. 2020. 33232044

[4] Frampton JE . Rituximab: a review in pemphigus vulgaris. Am J Clin Dermatol. 2020;21(1):149‐156. 31838645 10.1007/s40257-019-00497-9

[5] Schmidt E , Zillikens D . Pemphigoid diseases. Lancet. 2013;381(9863):320‐332. 23237497 10.1016/S0140-6736(12)61140-4

[6] Tavakolpour S , Mahmoudi H , Balighi K , Abedini R , Daneshpazhooh M . Sixteen‐year history of rituximab therapy for 1085 pemphigus vulgaris patients: a systematic review. Int Immunopharmacol. 2018;54:131‐138. 29132070 10.1016/j.intimp.2017.11.005

[7] Kamei K , Takahashi M , Fuyama M , et al. Rituximab‐associated agranulocytosis in children with refractory idiopathic nephrotic syndrome: case series and review of literature. Nephrol Dial Transplant. 2015;30(1):91‐96. 25085238 10.1093/ndt/gfu258

[8] Bielory L , Gascon P , Tj L , Ns Y , Mm F . Human serum sickness: a prospective analysis of 35 patients treated with equine anti‐thymocyte globulin for bone marrow failure. Medicine. 1988;67(1):40‐57. 3257288

[9] Maeda R , Kawasaki Y , Ohara S , Suyama K , Hosoya M . Serum sickness with refractory nephrotic syndrome following treatment with rituximab. CEN Case Rep. 2018;7:69‐72. 29305810 10.1007/s13730-017-0297-7 PMC5886928

[10] Aldarwish M , Almoosa Z . Serum sickness following tetanus toxoid injection. Case Rep Pediatr. 2021;2021:1‐3. 10.1155/2021/6680979 PMC7840246 33532106

[11] Pierpont TM , Limper CB , Richards KL . Past, present, and future of rituximab—the world's first oncology monoclonal antibody therapy. Front Oncol. 2018;8:163. 29915719 10.3389/fonc.2018.00163 PMC5994406

[12] Randall KL . Rituximab in autoimmune diseases. Aust Prescr. 2016;39(4):131‐134. 27756976 10.18773/austprescr.2016.053 PMC4993704

[13] Shetty S , Ahmed AR . Critical analysis of the use of rituximab in mucous membrane pemphigoid: a review of the literature. J Am Acad Dermatol. 2013;68(3):499‐506. 23200198 10.1016/j.jaad.2012.10.018

[14] Lamberts A , Euverman HI , Terra JB , Jonkman MF , Horváth B . Effectiveness and safety of rituximab in recalcitrant pemphigoid diseases. Front Immunol. 2018;9:335689. 10.3389/fimmu.2018.00248 PMC5827539 29520266

[15] Karmacharya P , Poudel DR , Pathak R , et al. Rituximab‐induced serum sickness: a systematic review. Paper presented at: Seminars in arthritis and rheumatism2015 2015. 10.1016/j.semarthrit.2015.06.014 26199061

[16] Bayer G , Agier M‐S , Lioger B , et al. Rituximab‐induced serum sickness is more frequent in autoimmune diseases as compared to hematological malignancies: a French nationwide study. Eur J Intern Med. 2019;67:59‐64. 31279430 10.1016/j.ejim.2019.06.009

[17] Khatib MY , Allafi SM , Nashwan AJ . Serum sickness following rituximab therapy in a patient with pemphigus vulgaris: a case report. Clin Case Reports. 2021;9(2):751‐754. 10.1002/ccr3.3642 PMC7869366 33598239

[18] Jolie Krooks B , Angela Weatherall MD . Rituximab‐induced serum sickness in pemphigus vulgaris: a case report and literature review. Pract Dermatol. 2019;(2):75‐78.

[19] Blase JR , Frame D , Michniacki TF , Walkovich K . Case report: use of obinutuzumab as an alternative monoclonal anti‐CD20 antibody in a patient with refractory immune thrombocytopenia complicated by rituximab‐induced serum sickness and anti‐rituximab antibodies. Front Immunol. 2022;13:863177. 35514985 10.3389/fimmu.2022.863177 PMC9061985

[20] Chakib C , Ayoub B , Achraf J , Khalil M , Hicham B . Rituximab induced acute respiratory distress syndrome (ARDS) reversed with plasmapheresis: case report. PAMJ‐Clinical Medicine. 2021;5(67):2‐6.

[21] Awal G , Kaur S , Kaur J , Sharma S . Rituximab‐induced serum sickness in pemphigus vulgaris. JEWDS. 2018;15(1):54‐56.

[22] Deshpande A . Delayed‐onset serum sickness due to rituximab in pemphigus vulgaris. Indian J Drugs in Dermatol. 2019;5(1):54.

[23] Podestà MA , Ruggiero B , Remuzzi G , Ruggenenti P . Case report: ofatumumab for multirelapsing membranous nephropathy complicated by rituximab‐induced serum‐sickness. BMJ Case Rep. 2020;13(1):e232896. 10.1136/bcr-2019-232896 PMC7035801 31980477

